# Selection for Oil Content During Soybean Domestication Revealed by X-Ray Tomography of Ancient Beans

**DOI:** 10.1038/srep43595

**Published:** 2017-02-27

**Authors:** Yunbing Zong, Shengkun Yao, Gary W. Crawford, Hui Fang, Jianfeng Lang, Jiadong Fan, Zhibin Sun, Yang Liu, Jianhua Zhang, Xiulan Duan, Guangzhao Zhou, Tiqiao Xiao, Fengshi Luan, Qing Wang, Xuexiang Chen, Huaidong Jiang

**Affiliations:** 1 State Key Laboratory of Crystal Materials, Shandong University, Jinan, Shandong 250100, China; 2 School of Physical Science and Technology, ShanghaiTech University, Shanghai 201210, China; 3 Department of Anthropology, University of Toronto Mississauga, Mississauga, Ontario, L5L 1C6, Canada; 4 Department of Archaeology, Shandong University, Jinan, Shandong 250100, China; 5 Shanghai Synchrotron Radiation Facility, Shanghai Institute of Applied Physics, Chinese Academy of Sciences, Shanghai 201800, China

## Abstract

When and under what circumstances domestication related traits evolved in soybean (*Glycine max*) is not well understood. Seed size has been a focus of archaeological attention because increased soybean seed weight/size is a trait that distinguishes most modern soybeans from their ancestors; however, archaeological seed size analysis has had limited success. Modern domesticated soybean has a significantly higher oil content than its wild counterpart so oil content is potentially a source of new insight into soybean domestication. We investigated soybean oil content using X-ray computed tomography (CT; specifically, synchrotron radiation X-ray CT or SRX-CT) of charred, archaeological soybean seeds. CT identified holes in the specimens that are associated with oil content. A high oil content facilitates the development of small holes, whereas a high protein content results in larger holes. The volume of small holes increased slowly from 7,500 to 4,000 cal B.P. We infer that human selection for higher oil content began as early as 7,500 cal B.P. and that high oil content cultivars were well established by 4,000 cal B.P.

Soybean (*Glycine max*) is one of the world’s most important crops largely because of its high protein (40%) and oil (20%) content^1^,^2^. Modern, domesticated soybean has significantly higher oil content than its wild/weedy counterpart (*G. max* subsp. *soja*, syn. *G. soja*)^3^,^4^,^5^ so selection for oil content probably played a role in soybean domestication. What the role may have been is not known. The Yellow River is acknowledged as an important area of agricultural evolution as well as plant and animal domestication during the Late Pleistocene and Early Holocene^6^ and appears to be one of at least three regions in East Asia where soybean was domesticated^7^. The earliest associations of people and soybean are in Early Holocene Neolithic communities in the region (9,000–7,500 cal B.P.)^7^,^8^,^9^,^10^,^11^,^12^. Cultivation also likely began at this time, when other crops such as millet and rice were being cultivated^13^. Domestication resulted in at least nine significant changes to the soybean phenotype and genotype^7^,^14^, among which seed size is the only trait so far accessible to archaeological examination. A study of archaeological soybean seed size points to a relatively late increase in seed size in China^8^. Thus, the selection processes that resulted in the traits that distinguish domesticated from wild soybean (*Glycine soja*, syn. *G. max* subsp*. soja*) today are poorly understood. For instance, soybean pods’ reduced dehiscence permits seed retention for harvesting while still allowing the seeds to be easily released during post-harvest processing^15^,^16^,^17^. Only one pod has been reported from an archaeological context in China but it has yet to be described^18^. Oil content has potential as an archaeologically visible trait that may help resolve when selection for domestication related traits in soybean began.

Research directed at soybean improvement that compares domesticated soybean variation with that of wild soybean to uncover the genetic foundation of productivity and nutritional content^19^,^20^ can inform the early domestication process. In particular, advances in omics, such as quantitative trait locus (QTL) mapping and genome-wide association studies (GWASs) are defining the genetic basis for soybean oil and protein variation^21^,^22^,^23^,^24^,^25^,^26^,^27^,^28^,^29^,^30^,^31^. In this report, we explore (1) X-ray computed tomography (CT) as a tool to examine a charred seed structure related to oil content and (2) how the results inform soybean domestication. CT was used to nondestructively reveal the interior three-dimensional structure of charred soybean seeds. CT scans of archaeological and modern control specimens indicate that a unique, porous structure in soybean comprised of non-randomly organized spaces that we call “holes” is related to oil content. The relationship also holds for protein because the oil and protein content of soybean seeds are negatively correlated. Archaeological, charred soybean seeds dating from 7,700 to 1,065 cal B.P. from the middle and lower Yellow River valley provide evidence that changes in oil content over time can be detected (Fig. 1). Furthermore, selection for higher levels of oil in soybean through time is evident.

The archaeological soybeans used in this study are from four sites on the North China Plain: Yuezhuang (YZ)^32^, Shenzhongji (SZJ)^33^, Daxinzhuang (DXZ)^34^ and Xijincheng (BXJ)^35^ (Fig. 1 and Supplementary Table S1), representing the Houli (B.P. 8,500–7,500), late Longshan (B.P. 4,200–3,900), Shang (B.P. 3,600–3,000) and Tang (B.P. 1,400–1,100) cultures. Soybean compositional traits are affected by environmental factors in addition to genetics so limiting this study to a narrow geographic region should minimize the impact of environmental differences on our study^25^,^36^,^37^.

A control sample of twenty-one seeds, seven from each of three land races of modern soybeans with known oil and protein content were CT-scanned to examine the relationship between the holes in the charred soybean seeds and their oil and protein content. The control samples were charred at 275 °C and 300 °C for 3 hours in order to better understand the impact of temperature and the resulting physical and chemical transformations affecting the structures of the archaeological specimens^38^. During the carbonization process, physical structure and chemical composition of seeds change as a function of temperature and heat treatment time^39^. Additionally, two charred, modern cultivated soybeans and one charred modern wild soybean were CT-scanned. Four other taxa with oil-rich seeds were also examined in order to determine whether oil content influences their structures after charring: hemp (*Cannabis sativa*), castor (*Ricinus communis*), perilla (*Perilla frutescens*), rapeseed (*Brassica napus*) and safflower (*Carthamus tinctorius*). These specimens were compared to archaeological seeds of four crops with low to no oil content but rich in starch or polysaccharides: bread wheat (*Triticum aestivum*), rice (*Oryza sativa*), buckwheat (*Fagopyrum esculentum*) and foxtail millet (*Setaria italica* subsp. *italica*). Finally, adzuki (*Vigna angularis*), a legume (Fabaceae) with low oil, high protein, and high starch content, rounded out the control sample.

Synchrotron radiation X-ray CT (SRX-CT), which was used in this study, is a non-destructive, three-dimensional imaging method used to obtain the three-dimensional or volumetric representation of an object. Synchrotron X-rays have higher flux and brightness than a conventional X-ray tube source, permitting higher resolution data to be collected. This method has proven effective in resolving problems with large palaeontological and archaeological specimens, such as fossil embryos^40^,^41^,^42^,^43^, Neanderthal molars^44^, and papyrus rolls recovered at Herculaneum^45^. To the best of our knowledge, this study represents the first time that valuable compositional information from charred seeds has been obtained using CT. Although the beam time limits the number of specimens that can be examined, we selected as many archaeological and modern soybeans and other crops as time permitted. Overall, thirty modern seeds, seven archaeological seeds, and ten other crop seeds were studied.

## Results

### Radiocarbon Dating

Accelerator mass spectrometer (AMS) radiocarbon dates were obtained for soybeans from each assemblage to confirm the ages of the archaeological beans (Table 1). The AMS dates confirm the chronological sequence; in addition, the soybeans are not intrusive into their respective contexts. The dates span a range of approximately 6,000 years.

### Holes

**Figure 2:**
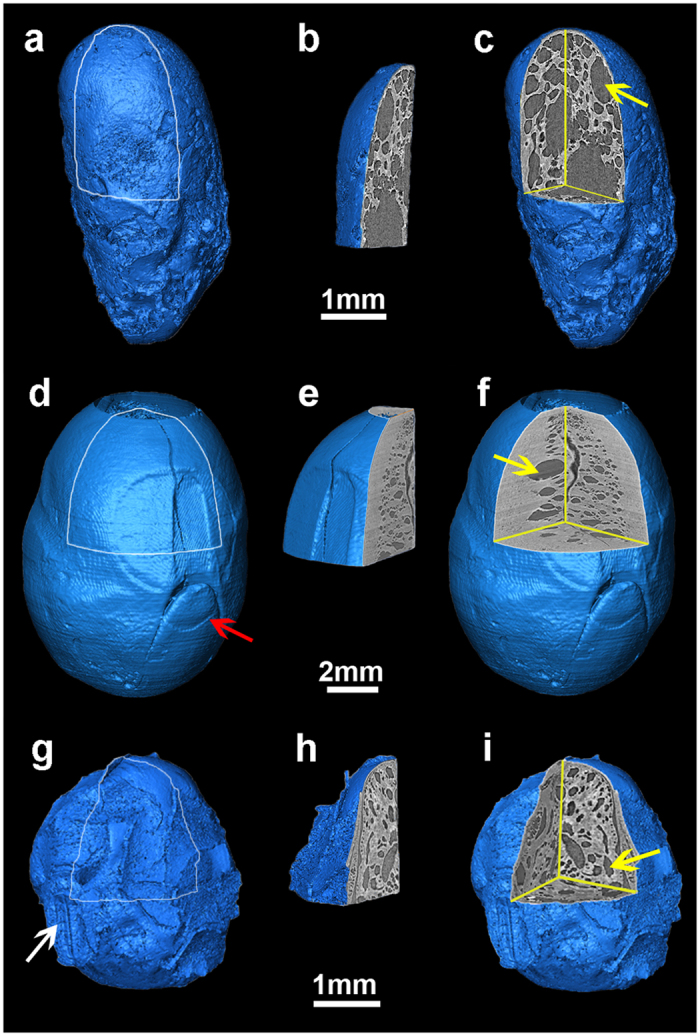
Three-dimensional reconstructed images of charred soybeans and their internal structures. (**a**,**d**,**g**) are the surface rendering results of archaeological soybean, modern cultivated soybean and modern wild soybean, respectively. (**b**,**e**,**h**) are segments cut from (**a**,**d**,**g**). (**c**,**f**,**i**) are the remaining portions. Little morphological information could be drawn from (**a**) because of the complicated physical, chemical and microorganism processes that resulted in significant degradation of the specimen. An embryo can be clearly recognized in (**d**), indicated by the red arrow. The hilum in (**g**) is indicated by the white arrow. (**g**) is a modern wild soybean collected locally. Holes in (**c**), (**f**) and (**i**) are indicated by the yellow arrows.

Charred soybean cotyledons almost never split apart and normally appear quite porous with numerous spaces or holes, rendering them unlike other charred bean family (Fabaceae) seeds recovered from archaeological sites in East Asia, such as adzuki (*V. angularis*). These spaces or holes are present in the cotyledons of each of the charred ancient, modern domesticated, and wild soybean specimens (Fig. 2a, d, and g, respectively). One spheroidal or ellipsoidal “hole” structure was discovered (yellow arrows in Fig. 2c,f and i). The sizes of the holes range from several tens of micrometers to hundreds of micrometers, which is substantially larger than the normal cotyledon cell size (Supplementary Fig. S1). The holes, therefore, are not specific cells.

### Hole Quantification

**Figure 3:**
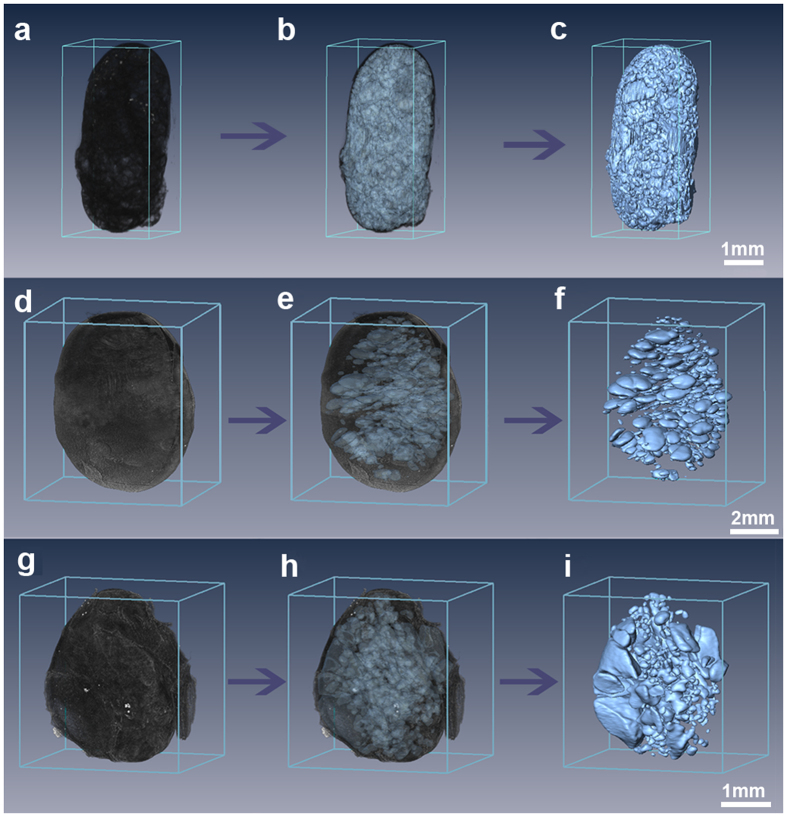
Segmentation of holes in 3D reconstructed images of soybeans. (**a**,**d**,**g**) are the volume rendering results of archaeological soybean, modern cultivated soybean and modern wild soybean, respectively, of the same seeds illustrated in Fig. 2. (**b**,**e**,**h**) are the transparent images showing holes in the soybeans. (**c**,**f**,**i**) are the surface results of the extracted holes.

The seeds were virtually segmented, and the hole volumes were calculated using Amira, a 3D visualization and analysis application (Mercury Computer Systems, Berlin, Germany). The three-dimensional segmentation result is displayed in Fig. 3. Holes that were too small to be differentiated were considered to be outliers and not included in the analysis. Large breaks that are a result of cotyledon separation were excluded. A few very small cracks are included because they could not be isolated from holes in the analysis. They represent less than 1 percent of the holes. The process of hole segmentation is shown in Supplementary Fig. S2 and indicates that holes are arranged throughout the cotyledons (Fig. 2), likely because the cotyledon cell structure is homogenous (Supplementary Fig. S3).

### Hole Formation

Tissue paraffin-sections were examined with a visible light microscope (Supplementary Fig. S1d) and transmission electron microscopy (Supplementary Fig. S3). Charring at high temperatures (~275 °C) clearly leads to hole generation in soybean seeds (Supplementary Fig. S1a–c). Holes are present in the five other oil-rich crops but they are more irregularly distributed than in soybean (Supplementary Fig. S4a–e). Holes were not detected in the charred, archaeological wheat, rice, millet and buckwheat seeds (Supplementary Fig. S5). Adzuki, with a low oil content, has only uniformly distributed holes (Supplementary Fig. S4f). The six control samples confirm that oil (very little to no oil is present in wheat, rice, millet and buckwheat) has a significant role to play in soybean hole formation. TEM imaging shows the distribution of protein and oil in the cells (Supplementary Fig. S3).

The process of hole formation appears to vary among the high oil content seeds, with soybean having a unique pattern, likely resulting from its particular oil and protein content. High temperatures cause complex chemical and physicochemical processes that ultimately cause the small oil droplets that are distributed throughout the seed to coalesce into larger drops^46^. Heating also changes the protein structure so that the seed is permeable to oil; that is, the oil can move through the cotyledons to coalesce and form pockets of oil that form the holes in the charred seeds. Once carbonized, the large oil droplets are eliminated from the charred soybeans^46^. Furthermore, the irregular distribution of protein bodies in soybean seeds may help explain the irregular structure noted in charred soybean cotyledons and not in crops rich in starch or polysaccharides^47^.

**Figure 4:**
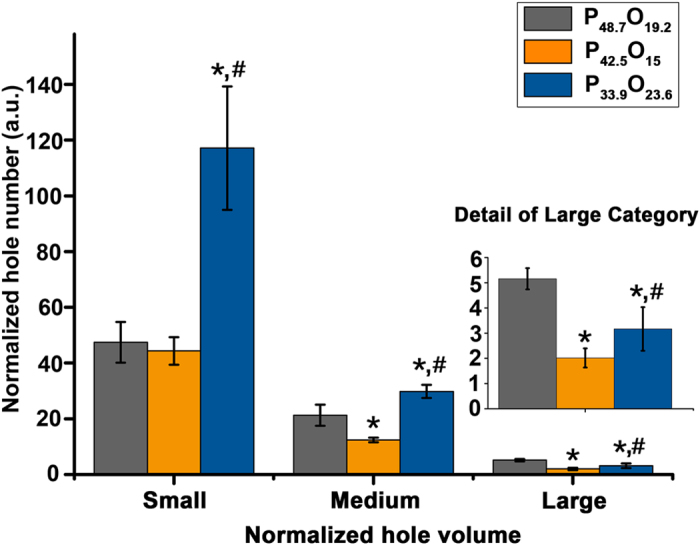
Statistics of normalized hole volume vs. normalized hole number of known protein and oil content soybeans. P_48.7_O_19.2_ (Meng), P_42.5_O_15_ (Fu), and P_33.9_O_23.6_ (Tiefeng) are three different cultivars of soybeans with varying protein and oil content. Seven seeds were averaged for each cultivar. The asterisk (∗) indicates significant differences compared with holes in P_48.7_O_19.2_ (*P* < 0.05; one way ANOVA test). The pound sign (#) indicates significant differences compared with holes in P_42.5_O_15_ (*P* < 0.05; one way ANOVA test). The bars indicate standard error. Statistically not significant differences with *P* > 0.05 are not indicated.

To further assess how protein and oil affect the generation of holes, cultivated soybean seeds were examined. First, the protein and oil content was measured^48^,^49^,^50^. Then, the specimens were carbonized at 275 °C for 3 hours. We selected three groups of soybeans whose protein and oil content varied: Meng (protein 48.7% and oil 19.2%, P_48.7_O_19.2_), Fu (protein 42.5% and oil 15%, P_42.5_O_15_) and Tiefeng (protein 33.9% and oil 23.6%, P_33.9_O_23.6_). Seven seeds in each group were CT-scanned. The normalized hole numbers and normalized hole volumes (absolute hole counts and volumes were divided by the soybean volume to calculate normalized values and are hereafter referred to simply as “volume” and “number.”) were used for statistics (Fig. 4). We then split the volumes into three categories using Amira: Small (1E-7 order), Medium (1E-6 order) and Large (1E-5 order). There were more holes in P_33.9_O_23.6_ than in P_48.7_O_19.2_ or P_42.5_O_15_ in the Small category (*P* = 0.0002, one-way ANOVA and Dunnett’s T3 analysis for both, Fig. 4). In the Medium category, P_33.9_O_23.6_ and P_48.7_O_19.2_ had more holes than P_42.5_O_15_ that had the lowest oil content (*P* < 0.0001 and *P* = 0.002, respectively, one-way ANOVA and Dunnett’s T3 analysis for both). However, in the Large category, P_48.7_O_19.2_ a had a greater mean than P_42.5_O_15_ or P_33.9_O_23.6_ due to its higher protein content (*P* < 0.0001 for both, one-way ANOVA and Fisher’s LSD *post hoc* analysis for both). Regardless, this was not the case for the medium protein content of P_42.5_O_15_ compared with P_33.9_O_23.6_ (*P* = 0.002, one-way ANOVA and Fisher’s LSD *post hoc* analysis). In summary, the comparison of P_48.7_O_19.2_ with P_42.5_O_15_ indicates that the overall protein and oil content is directly correlated with hole number. Considering the extremely high oil content of P_33.9_O_23.6,_ we conclude that oil facilitates the generation of smaller holes. In turn, a higher protein content leads to the generation of larger holes.

### Temperature Gradient Experiment

**Figure 5:**
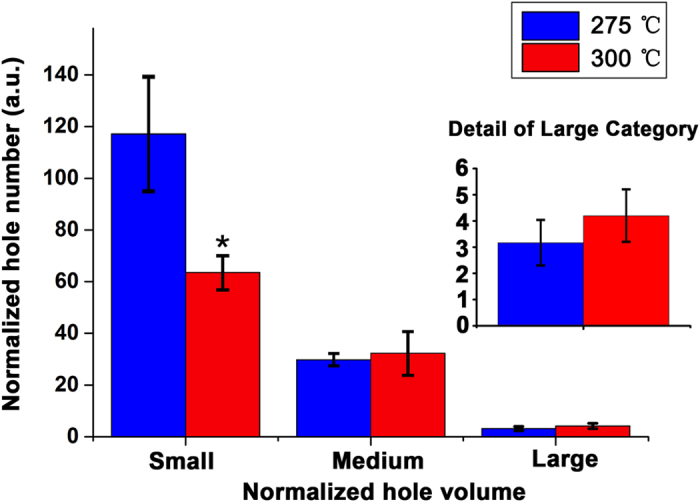
Temperature gradient experiment. Six P_33.9_O_23.6_ soybean seeds were carbonized at 275 °C. In the Small category, seeds charred at 300 °C had about half the number of seeds with small holes then when fired at 275 °C. The Medium and Large categories were not affected by temperature. The asterisk (∗) indicates significant differences compared with holes in 275 °C (*P* < 0.01, two-tailed t-test). The bars indicate standard error. Statistically not significant differences with *P* > 0.05 are not indicated.

A sample of six of the P_33.9_O_23.6_ seeds were charred at 300 °C for 3 hours. The number of holes in the Small category (*P* = 0.0004, two-tailed t-test, Fig. 5), was less at 300 °C than at 275 °C and little difference was noted in the Medium and Large categories (*P* = 0.51 and *P* = 0.07, two-tailed t-test for both). In summary, hole number in the Small category clearly reduced when the charring temperature increased from 275 °C to 300 °C.

### Water Saturation Experiment

**Figure 6:**
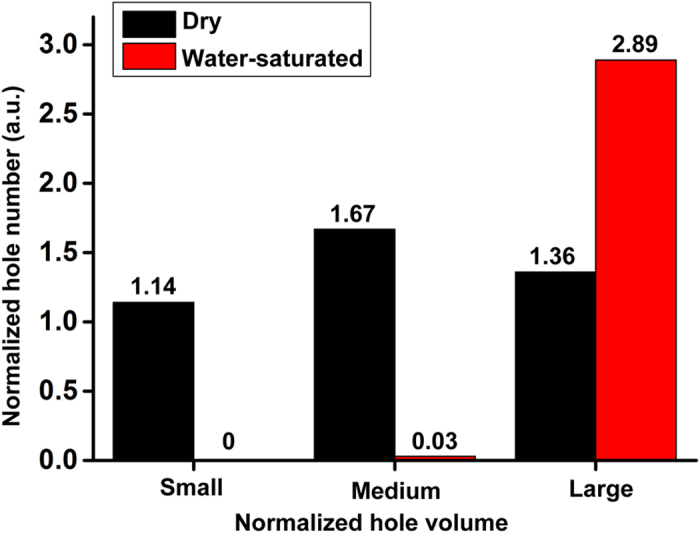
Water saturation experiment. A water-saturated soybean seed was charred and CT-scanned. Normalized hole volumes were split into three categories: Small (1E-7 order), Medium (1E-6 order) and Large (1E-5 order). Compared with one dry seed of the same cultivar, few holes (below 1E-5) were detected in the water-saturated seed.

Soybean seeds readily absorb water so we investigated the potential role of water in hole formation. Modern seeds of the unnamed cultivar (shown in Fig. 2d) were soaked for 10 hours and then charred at 275 °C for 3 hours. After soaking, the beans enlarged approximately 1.4 times their original size. After charring, the soaked beans were less dense and more brittle than the unsoaked beans. One charred seed was selected for CT scanning. We calculated the total hole volume, the post-charring mass density and other relevant parameters (Supplementary Table S2). The holes became larger compared with the dry seed due to soaking in water and smaller holes (volume level below 1E-5) were not evident (Fig. 6). The hole structure of charred, water-saturated seeds does not resemble any of the archaeological specimens, so water can be eliminated as a significant factor impacting the hole structure of the archaeological samples in this study.

## Discussion

**Figure 7:**
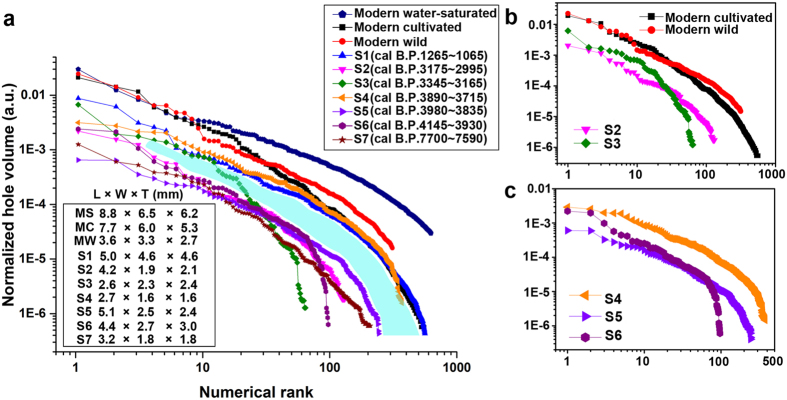
Quantification analysis of soybean holes. (**a**) Seven archaeological soybeans from different periods and three modern soybeans are compared with each other and with modern water-saturated (MS), modern cultivated (MC) and modern wild (MW) soybeans. The blue shading separates the data into two: modern cultivated and wild soybean and archaeological soybeans S1 and S4 in the upper region, and others in the lower region. (**b**) Compares modern cultivated and wild soybeans, S2 and S3. The negative correlation between the oil and protein content of S2 and S3 is indicated. Modern cultivated soybean has a higher oil content than the modern wild seed. (**c**) Three Longshan period soybeans: S4–6. A negative correlation between the oil and protein content is indicated in S5 and S6. However, S4 shows an unusual distribution.

CT scanning revealed a particular hole structure in charred seeds that we linked to oil and protein content. The hole distribution for each bean is presented as the normalized hole volume plotted against numerical rank (Fig. 7). Numerical rank represents the holes in each bean sorted from large to small. Each curve represents one soybean, and each symbol on a curve indicates one hole. Seven archaeological soybeans and three modern soybeans are shown (Fig. 7). The number of holes may be less important than the size distribution of holes because the complete hole structure of many seeds is masked by their incompleteness.

Oil percentage has tended to increase through time (Fig. 7a). Three modern soybean seeds (two domesticated and one wild) and two archaeological soybean seeds from the Tang dynasty (S1) and Longshan period (S4) have significantly higher hole volumes than the others (Group 1). Beans from the Shang (S2, S3), Longshan (S5, S6) and Houli periods (S7) have a lower hole volume distribution (Group 2). The modern cultivated soybean has significantly more oil than modern wild soybean (Fig. 7b), so the earliest soybean seeds in the archaeological record, if wild, should have also had low oil content. All archaeological specimens in this study appear to have substantially higher oil content than that of the modern wild soybean seed.

A comprehensive analysis of archaeological soybean seed size indicated that both wild/weedy and domesticated soybean seeds are represented in the archaeological record at sites dating to the Longshan and Shang periods in China^7^. We compared the protein and oil content of the two size modes at the Shang period Daxinzhuang site (groups S2 and S3; Fig. 7b) in order to test whether there are significant differences in the seed composition of the two size categories. Their size difference is distinct, with S2 averaging (L, W, T) 4.2 by 1.9 by 2.1 mm and S3 2.6 by 2.3 by 2.4 mm. The previous study suggested that S3 was wild/weedy and that S2 was cultivated/domesticated^7^. S3 (hypothetically wild/weedy) has a lower percentage of oil but higher percentage of protein than S2. S2 (hypothetically domesticated soybean) has a higher percentage of oil and lower protein percentage than S3. The results are consistent with S2 being cultivated/domesticated and S3 being wild/weedy.

S5 and S6 are from the Longshan period Xijincheng site. The specimens both fall within the large mode although some size differentiation is evident. S5 is longer than S6 (5.1 by 2.5 by 2.4, product = 30.6) vs. (4.4 by 2.7 by 3.0, product = 35.6). Though differentiation in the hole number range 20–60 is similar, there are more small holes after number 80 in S5 that has a higher oil percentage than S6 (Fig. 7c).

The archaeological record for soybean in China indicates that seed size increase was a late development and that the size increase when it did occur was not comparable to the changes in soybean size documented in the Japanese and Korean archaeological record^7^. Thus, in China, traits other than seed size were probably being selected during the Early through Late Neolithic. If high oil content was being selected, then oil content would increase and not necessarily be directly correlated with size during that period. This appears to be the case. The Longshan S4 sample is quite small (2.7 by 1.6 by 1.6 mm) compared to S5 and S6 yet has higher protein and oil content (Fig. 7c). However, due to the lack of large-sized seeds from the same context, the hole distribution in S4 is difficult to explain. The Houli seed (S7), the oldest specimen considered in this study, has a higher oil content than the modern wild soybean seeds. We suggest that the Houli soybeans had already undergone some selection for higher oil content by 7,500 B.P.

The distribution of smaller holes indicates that oil content significantly increased relative to protein content as soybean was undergoing domestication in China. The light-blue region (Fig. 7a) divides Group1 from Group 2, indicating a wide chronological gap during which selection proceeded. The Houli period Yuezhuang sample (S7) suggests that selection under cultivation had begun at least 7,500 years ago and that soybean domestication spanned a period of at least 3,500 years and probably longer. A larger sample from the period spanning 8,000–4,000 B.P. is needed to fully test this hypothesis.

To compare the control soybeans (P_48.7_O_19.2_, P_42.5_O_15_ and P_33.9_O_23.6)_ with the archaeological seeds (S1-S7), a similar plot of hole distribution is displayed in Supplementary Fig. S6. Before hole number 2500, P_48.7_O_19.2_ and P_42.5_O_15_ are located an upper position due to their high content of protein (48.7% and 42.5%). After hole number 2500, P_33.9_O_23.6_ surpasses P_48.7_O_19.2_ and P_42.5_O_15_ due to its high oil content.

## Conclusions

The effectiveness of CT scanning in investigating plant domestication was tested on a sample of archaeological soybeans from four archaeological sites in the Yellow River valley spanning the period from 7,500 to 1,065 cal B.P. A “hole” structure in charred soybean seeds appears to be linked to protein and oil content. We hypothesize that smaller holes are related to oil content and larger holes are related to protein content. Selection for seed composition appears to have occurred as early as 7,500 B.P., indicating that soybean domestication and cultivation were occurring by this time. Significant differentiation of soybean is not evident until 4,000 B.P., and by 3,500 B.P., during the Shang period, soybean seed size and oil content substantially increased. Soybean likely underwent further selection for large seed size and higher oil and protein content, distinguishing it further from its wild/weedy ancestor that had smaller seeds, less oil, and higher protein^25^. Importantly, the “hole” structure is a new clue that helps our understanding of soybean domestication; without CT, this structure could not have been assessed or quantified. CT offers significant potential for archaeologists and biologists exploring plant domestication.

## Methods

### Soybeans and other crop seeds

All archaeological seeds were provided by the Department of Archaeology, Shandong University. The reference soybeans were provided by Institute of Crop Sciences at the Chinese Academy of Agricultural Sciences (ICS-CAAS). The wild soybeans were provided by the Shandong Academy of Agricultural Sciences. The other modern domesticated crop seeds were purchased from a local seed supplier.

### Carbonization experiment

A laboratory muffle furnace was used to char the modern seeds. The seeds were baked for 3 hours under anoxic conditions.

### X-Ray computed tomography experiment and 3D reconstruction

The tomography experiment was conducted on beamline 13W1 at the Shanghai Synchrotron Radiation Facility (SSRF) (Supplementary Fig. S7). The Wiggler system provides high monochromaticity of ΔE/E < 5 × 10^−3^. For specimens of different sizes, an X-ray energy range of 12–15 keV was selected. A charge coupled device (CCD) detector of a pixel size of 6.5 μm, with two interchangeable lenses - 2 × and 4 × magnification, were used for the experiment. Thus, the effective pixel resolution can be respectively reduced to 3.25 μm and 1.625 μm for tomographic images. To enhance the projection contrast, that is, to make full use of both absorption contrast and phase contrast, we set the distance between samples and CCD as 8 cm. The in-line phase contrast tomography improved contrast compared with conventional absorption contrast tomography^51^,^52^. Flat images were collected when the sample moved out of the beam path. Finally, when all of the projections and flat images had been collected, the shutter was switched off to shut down the X-ray beam, and five dark images were taken in order to subtract the dark noise. Then, parts of the images were used to reconstruct the three-dimensional result using equally sloped tomography (EST)^53^. The others were reconstructed using a filtered back projection (FBP) algorithm^54^ with the PITRE software^55^. All details regarding the scanning parameters and radiation dose for each seed are listed in Supplementary Table S3.

### Paraffin sectioning and electron microscopy experiment

The soybean tissue paraffin-sectioning was performed in reference to standard plant paraffin section flow^56^. Seeds underwent electron microscopy using a general laboratory biology electron microscope. Protein and oil were identified by staining.

### Statistical analysis

All statistical analyses were performed using Predictive Analysis Software 18.0 (SPSS Inc., Chicago, IL, USA). Intergroup comparisons were performed using one-way ANOVA followed by the LSD test (with equal variances assumed) or Dunnett’s T3 test (equal variances not assumed). One-way ANOVA was used to compare the three soybean cultivars. A two-tailed t-test was used to compare two groups of P_33.9_O_23.6_ that were charred at different temperatures.

### Data availability

The data that support the findings of this study are included in this article and in the Supplementary Information Files, or are available from the corresponding authors upon request.

## Additional Information

**How to cite this article:** Zong, Y. *et al*. Selection for Oil Content During Soybean Domestication Revealed by X-Ray Tomography of Ancient Beans. *Sci. Rep.* **7**, 43595; doi: 10.1038/srep43595 (2017).

**Publisher's note:** Springer Nature remains neutral with regard to jurisdictional claims in published maps and institutional affiliations.

## Supplementary Material

Supplementary Information

## Figures and Tables

**Figure 1:**
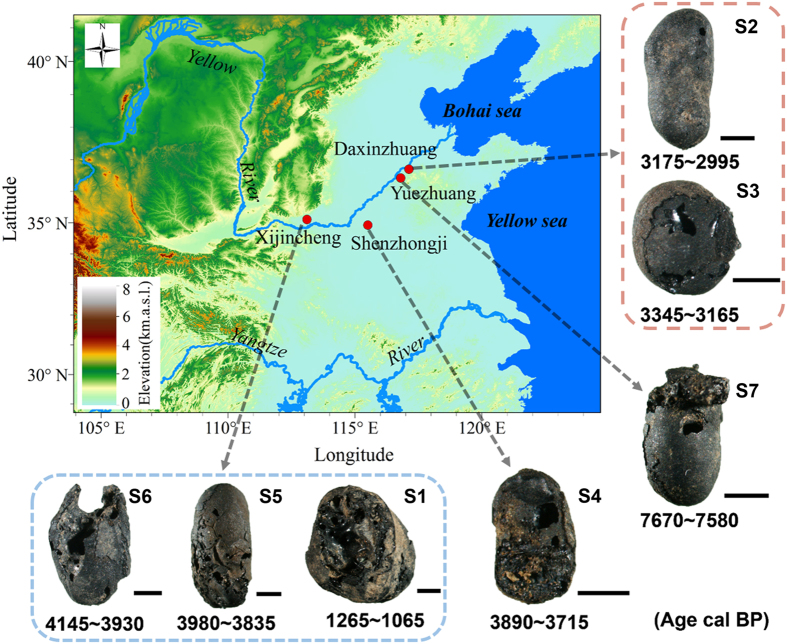
Location of sites from which CT-scanned archaeological soybeans were recovered. The numbers below each soybean image indicate AMS dates (cal B.P., 2- sigma range). Scale bar: 1 mm. The map was created with ArcGIS v.10.1 (ESRI, http://www.esri.com/software/arcgis) and annotated by the authors.

**Table 1:** Direct AMS radiocarbon dates of archaeological charred soybean seeds.

Sample number	Site name	Culture/Period	Radiocarbon Lab number (all Beta)	Conventional ^14^C age B.P.	2 Sigma calibrated (cal B.P.)
S1	Xijincheng	Tang	418894	1,230 ± 30	1,265–1,065
S2	Daxinzhuang	Shang	418904	2,940 ± 30	3,175–2,995
S3	Daxinzhuang	Shang	418900	3,040 ± 30	3,345–3,165
S4	Shenzhongji	Longshan	418895	3,530 ± 30	3,890–3,715
S5	Xijincheng	Longshan	418903	3,600 ± 30	3,980–3,835
S6	Xijincheng	Longshan	418898	3,690 ± 30	4,145–3,930
S7	Yuezhuang	Houli	418897	6,820 ± 40	7,700–7,590

The half-life of ^14^C is 5,568 y. CT-scanned S1, S3, and S5–6 were dated. S2, S4 and S7 used both the scanned and associated soybean seeds from the same archaeological feature to enable sufficient carbon for dating.
