# Supplementary material for: Selection for Oil Content During Soybean Domestication Revealed by X-Ray Tomography of Ancient Beans
Source: Sci Rep. 2017 Feb 27;7:43595. doi: 10.1038/srep43595 (PMC5327410; doi:10.1038/srep43595)
Supplement: Supplementary Information [file srep43595-s1.pdf]

Supplementary Information

Selection for Oil Content During Soybean Domestication Revealed by X-Ray Tomography of Ancient Beans

Yunbing Zong[1], Shengkun Yao[2], Gary W. Crawford[3], Hui Fang[4], Jianfeng Lang[4], Jiadong Fan[2], Zhibin Sun[1], Yang Liu[1], Jianhua Zhang[1], Xiulan Duan[1], Guangzhao Zhou[5], Tiqiao Xiao[5], Fengshi Luan[4], Qing Wang[4], Xuexiang Chen[4*], Huaidong Jiang[1,2*]

[1]State Key Laboratory of Crystal Materials, Shandong University, Jinan, Shandong 250100, China

[2]School of Physical Science and Technology, ShanghaiTech University, Shanghai 201210, China

[3]Department of Anthropology, University of Toronto Mississauga, Mississauga, Ontario, Canada, L5L 1C6

[4]Department of Archaeology, Shandong University, Jinan, Shandong 250100, China

[5]Shanghai Synchrotron Radiation Facility, Shanghai Institute of Applied Physics, Chinese Academy of Sciences, Shanghai 201800, China

[*]***Corresponding authors:***

Name: Huaidong Jiang

Address: 393 Middle Huaxia Road, ShanghaiTech University, Shanghai, China.

Telephone number: +86 21-20685300

Email address: jianghd@shanghaitech.edu.cn

Name: Xuexiang Chen

Address: Shanda Nanlu 27, Shandong University, Jinan, China.

Telephone number: +86 13675316943

Email address: xxchen@sdu.edu.cn

Supplementary Figures

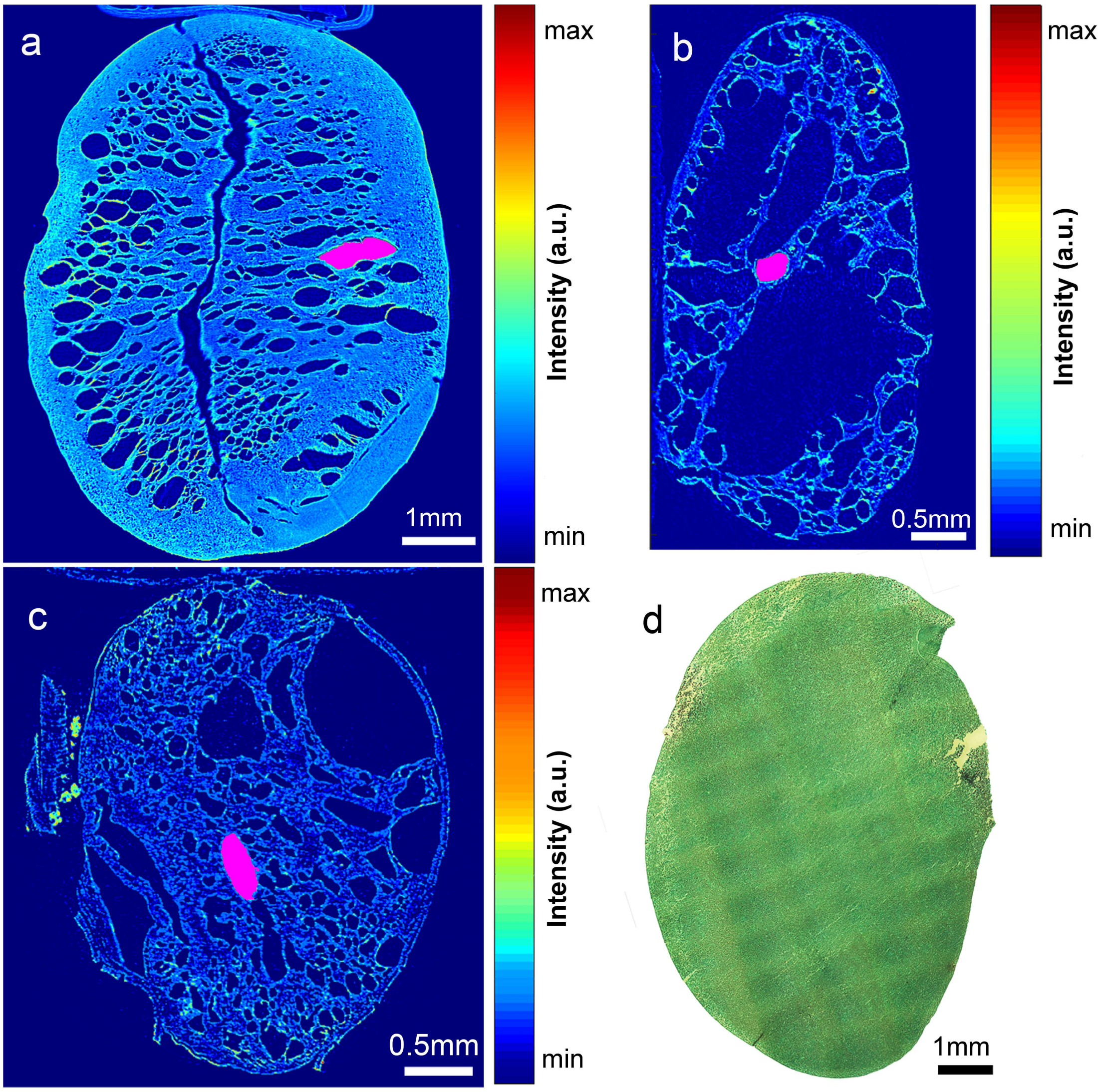

Figure S1 | Sections through charred soybeans. (**a**) Modern cultivated soybean; (**b**) archaeological soybean; (**c**) modern wild soybean; (**d**) modern uncharred, cultivated soybean paraffin section. Magenta regions show holes inside slices (**a**), (**b**) and (**c**). No holes are present in the fresh soybean (**d**).

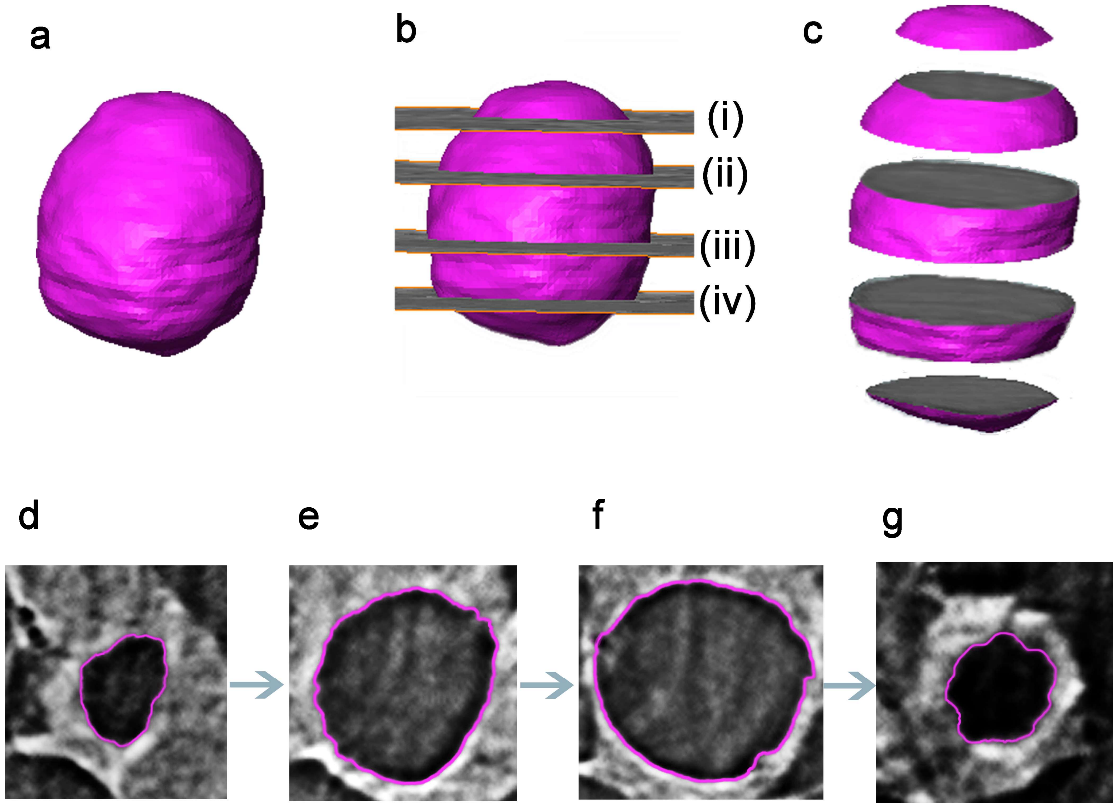

Figure S2 | Hole segmentation process for 3D images. (**a**) Single hole inside soybean cotyledon, (**b**)four evenly spaced slices, (**c**) segmented hole and (**d-g**) cross-sections of the hole; the sections correspond to the slices labelled i-iv in (**b**).

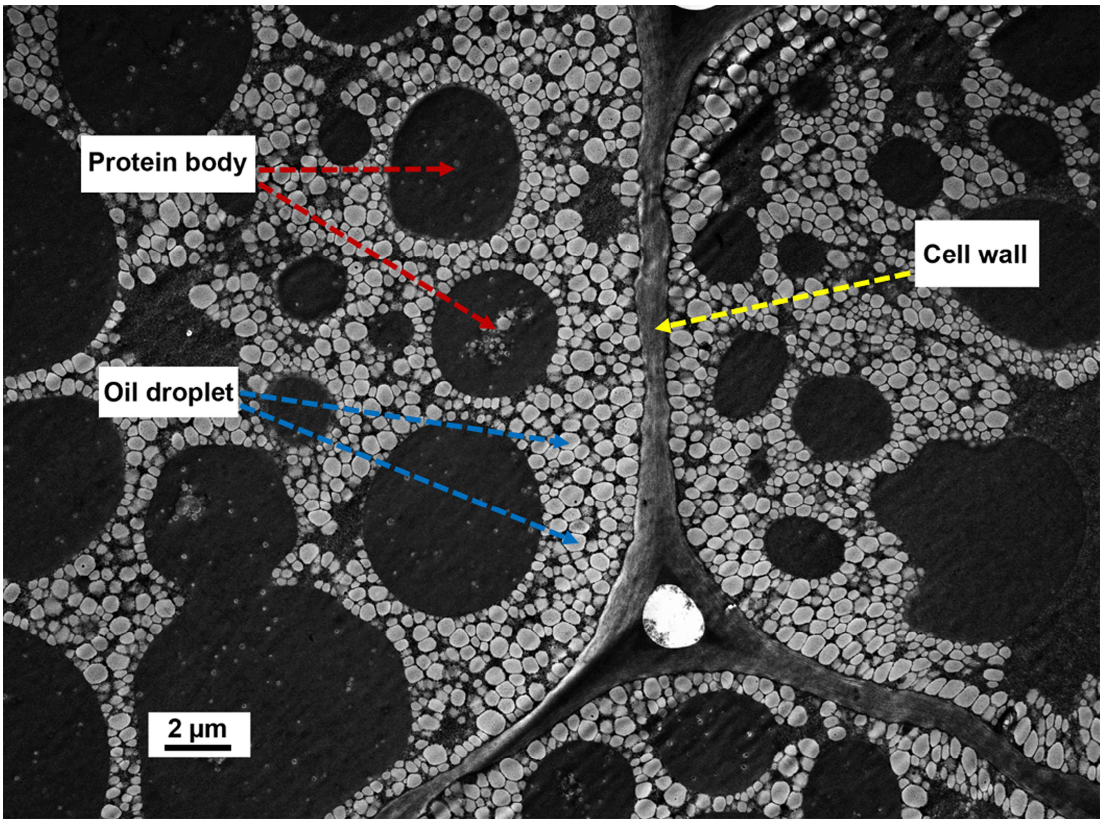

Figure S3 | TEM image of fresh soybean section. Black circles are protein bodies and the smaller white particles are oil droplets. Notably, the size of holes in charred soybeans is substantially great than size of the protein bodies.

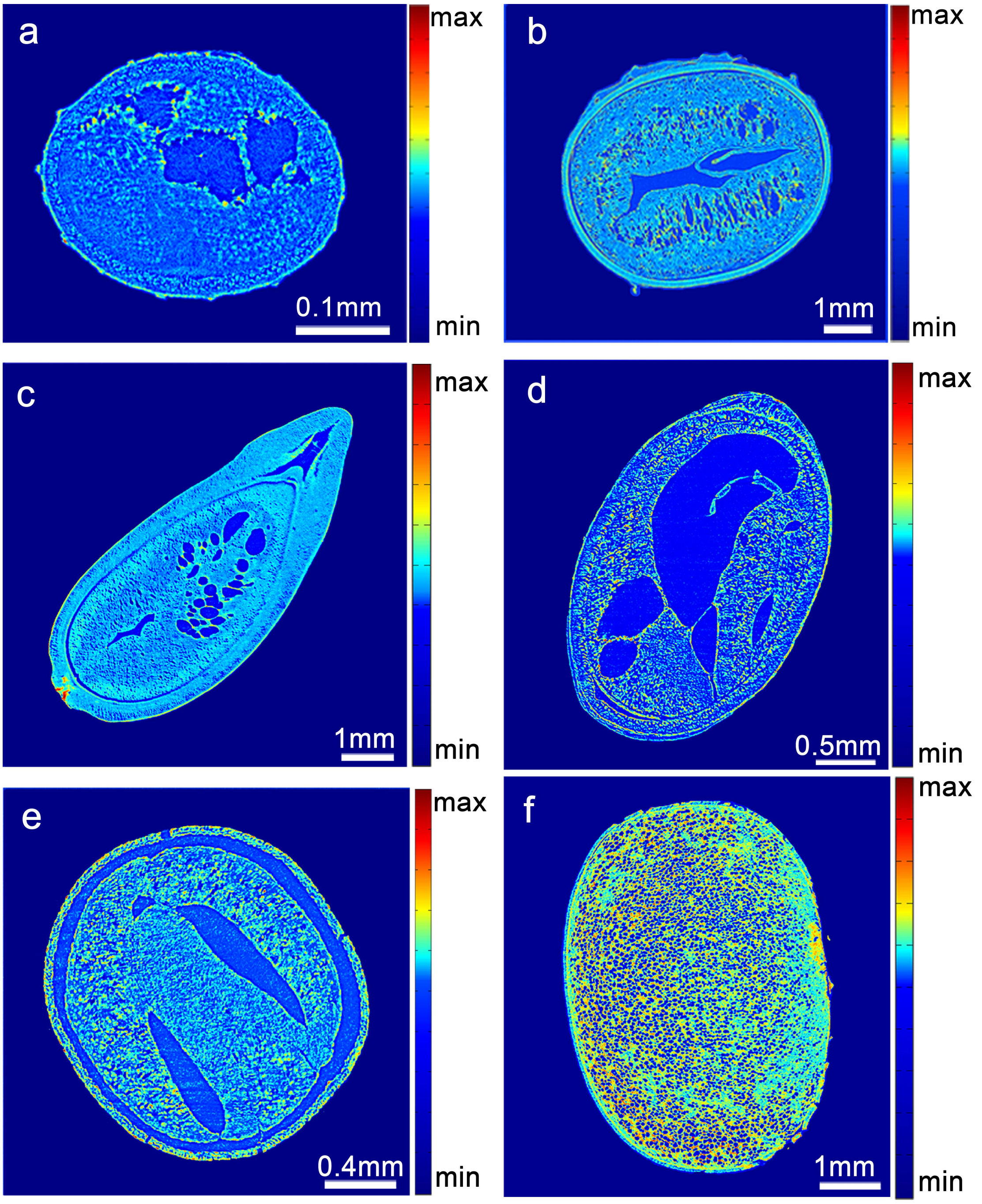

Figure S4 | Virtual sections of modern common crops rich in oil and not. (**a**) perilla (*Perilla frutescens*), (**b**) castor (*Ricinus communis*), (**c**) safflower (*Carthamus tinctorius*), (**d**) hemp (*Cannabis sativa*), (**e**) rapeseed (*Brassica napus*), (**f**) adzuki bean (*Vigna angularis*).

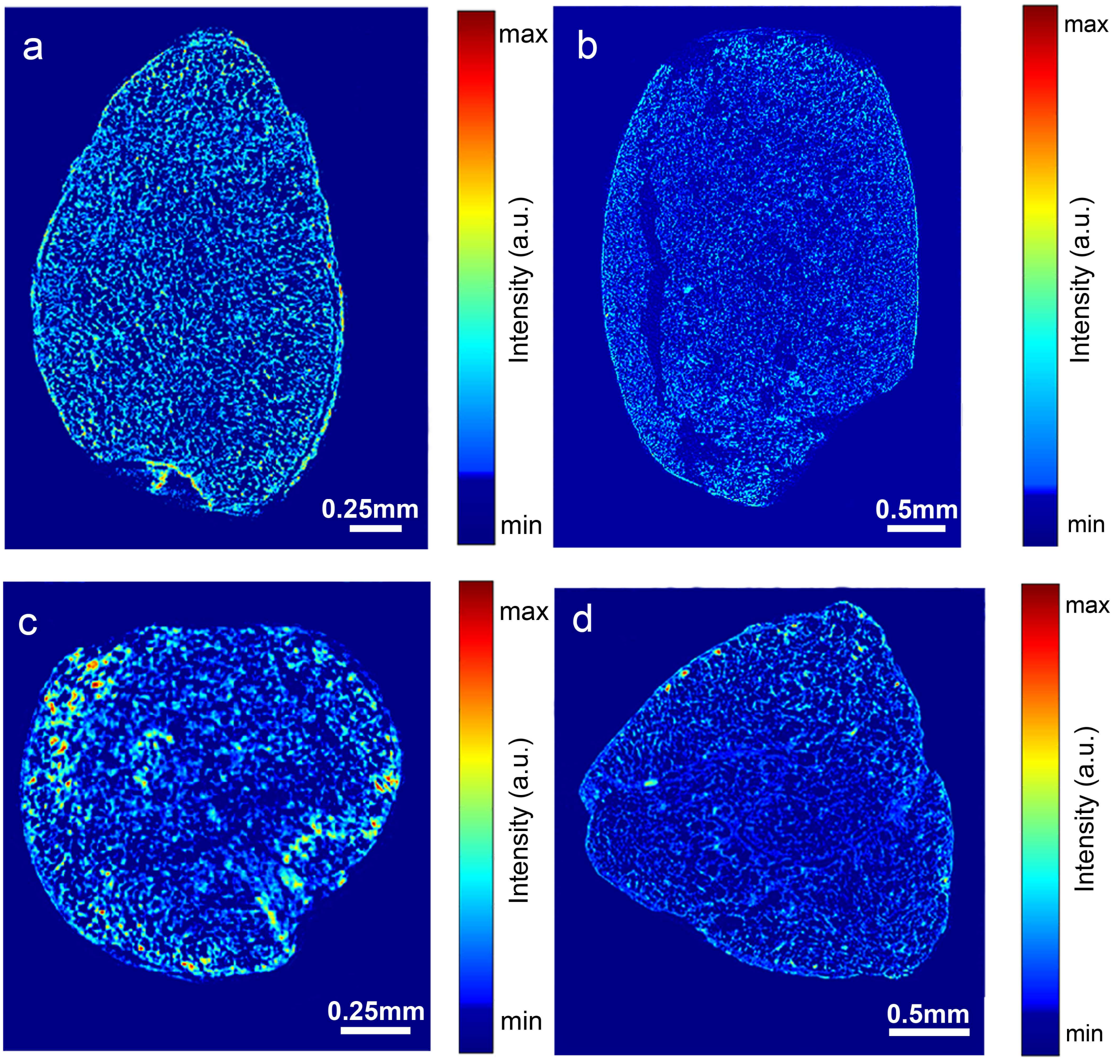

Figure S5 | Virtual sections of other archaeological crops: (**a**) bread wheat (*Triticum aestivum*), (**b**) rice (*Oryza sativa*), (**c**) foxtail millet (*Setaria italica* subsp. *italica*), and (**d**) buckwheat (*Fagopyrum esculentum*).

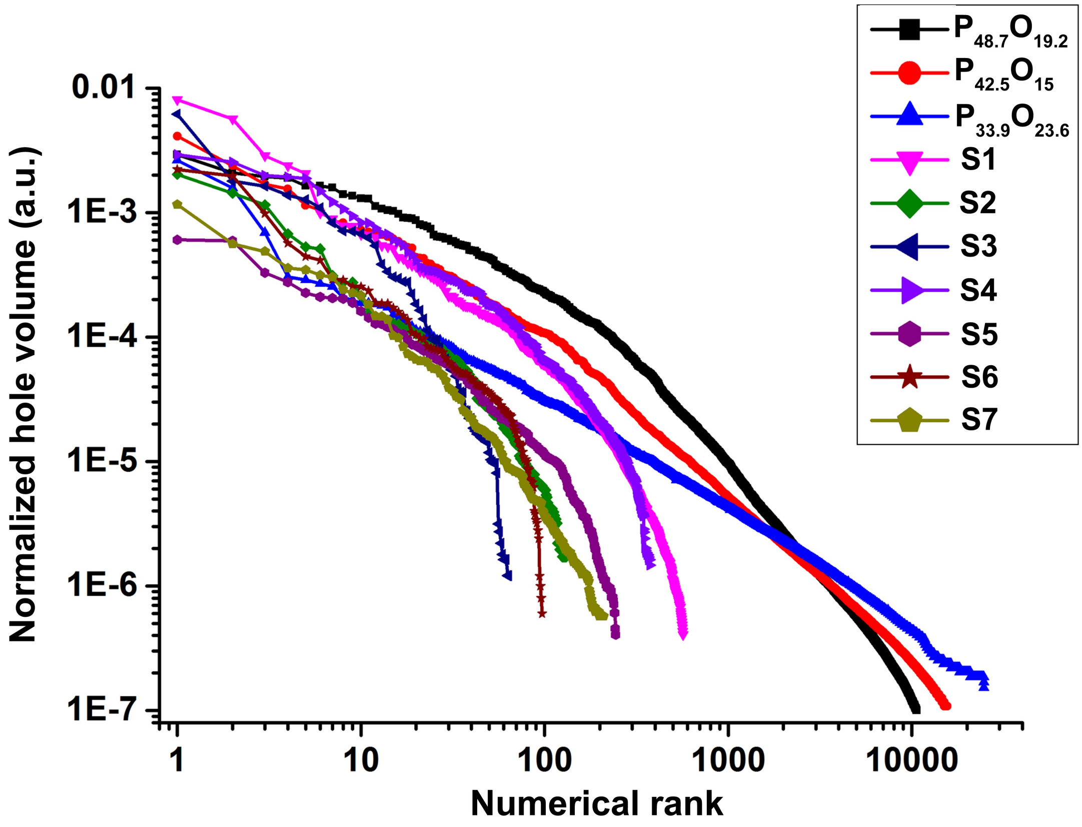

Figure S6 | Quantification analysis of holes of known protein and oil content soybeans and the archaeological seeds. Each line represents one seed. Three representative seeds from $P_{48.7}O_{19.2}$, $P_{42.5}O_{15}$ and $P_{33.9}O_{23.6}$ were plotted.

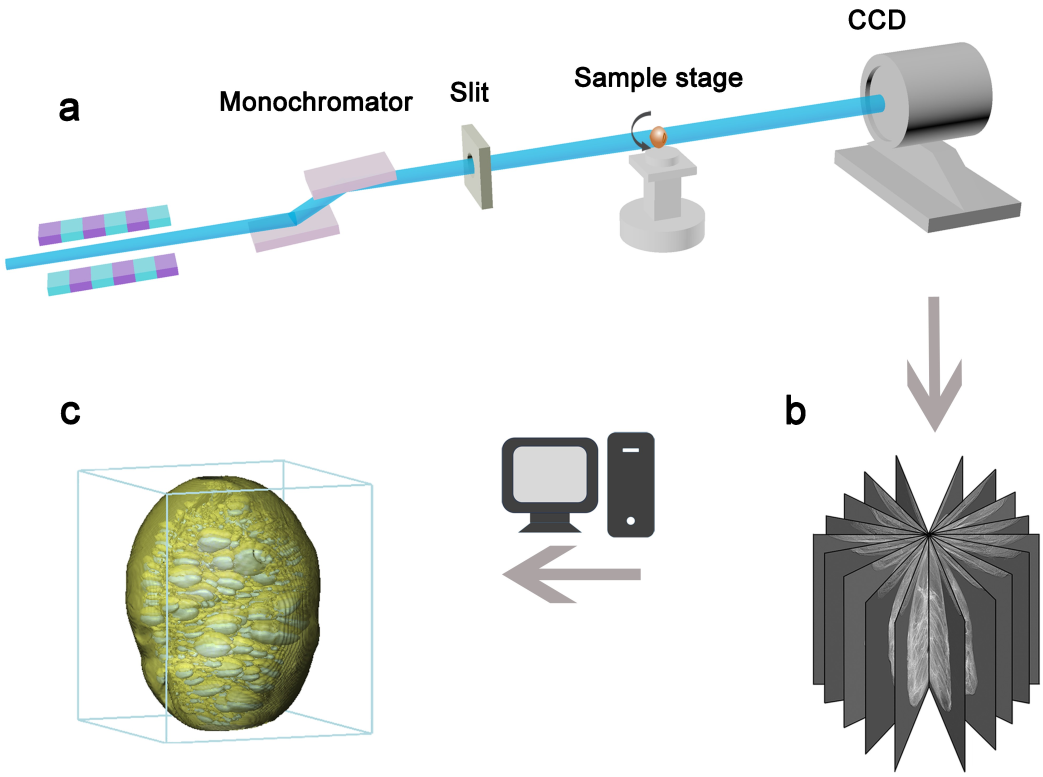

Figure S7 | Noninvasive X-ray computed tomography (CT) experiment. (a) Schematic showing synchrotron X-ray tomography at the Shanghai Synchrotron Radiation Facility; (**b**) Stereo view of different cross projection images of a soybean seed; (**c**) virtually reconstructing the 3D soybean by combining computer projections with a reconstruction algorithm.

Supplementary Tables

Supplementary Table S1. Culture chronology for the North China Plain[1,2].

Culture/Period	Dating (BC)
**Bronze Age**	
Eastern Zhou	ca. 770-221
Western Zhou	ca. 1,046-771
Middle and Late Shang	ca. 1,400-1,046
Yueshi culture	ca. 1,800-1,400
Erlitou culture	ca. 1,850–1,550
**Neolithic**	
Longshan culture	ca. 2,600–1,900
Dawenkou culture	ca. 4,150-2,650
Beixin culture	ca. 5,000-4,100
Houli culture	ca. 6,550-5,550

1. Underhill, A. P. *A Companion to Chinese Archaeology* (ed. Underhill, A. P.) (Wiley, 2013).
2. Liu, L., & Chen, X. *The Archaeology of China: From the Late Paleolithic to the Early Bronze Age* (Cambridge University Press, 2012).

Supplementary Table S2. Experiment to compare the effects of charring on a water-saturated soybean seed and a dry seed.

Characteristics	Dry	Water Saturated
Weight (g)	0.11	0.12
Volume (mm^3)	131.76	214.63
Total number of holes	562	787
Total volume of holes (mm^3)	20.55	51.38
Total volume ratio of holes (%)	15.6	23.9
Post-charring volume (mm^3)	111.21	163.25
Post-charring density (mg/mm^3)	0.99	0.74

Supplementary Table S3. CT experiment details for all samples.

Samples	seed number	projection number	energy/keV	pixel size/μm	exposure time/s	Radiation dose/kGy	method
modern soaked soybean	1	1200	13	6. 5	0. 0035	0. 1	FBP
modern cultivated soybean	1	1500	13	3. 25	1	10	FBP
modern wild soybean	1	900	12	3. 25	1	4	FBP
$P_{48.7}O_{19.2}$ (Meng)	7	1080×4, 1260×3	15	6. 5	0. 009	0. 1, 0. 2	FBP
$P_{42.5}O_{15}$ (Fu)	7	1400×3, 1260×4	15	6. 5	0. 009	0. 2, 0. 2	FBP
$P_{33.9}O_{23.6}$ (Tiefeng), 275ºC and 300ºC	13	1080×7, 1260×6	15	6. 5	0. 009	0. 1, 0. 2	FBP
ancient soybean (S1)	1	1400	15	3. 25	1	3	FBP
ancient soybean (S2)	1	540	12	3. 25	1	2	FBP
ancient soybean (S3)	1	540	12	3. 25	1	2	FBP
ancient soybean (S4)	1	400	12	3. 25	1	2	FBP
ancient soybean (S5)	1	1400	12	3. 25	1	6	FBP
ancient soybean (S6)	1	650	12	3. 25	1	2	FBP
ancient soybean (S7)	1	540	12	3. 25	1	2	FBP
ancient bread wheat (*Triticum aestivum*)	1	200	12	3. 25	1	1	EST
ancient rice (*Oryza sativa*)	1	600	12	3. 25	1	3	FBP
ancient foxtail millet (*Setaria italica* subsp. *italica*)	1	320	12	3. 25	1	2	FBP
ancient buckwheat (*Fagopyrum esculentum*)	1	620	12	3. 25	1	1	FBP
modern perilla (*Perilla frutescens*)	1	640	12	1. 625	5	1	FBP
modern castor (*Ricinus communis*)	1	1000	12	6. 5	0. 01	0. 1	FBP
modern safflower (*Carthamus tinctorius*)	1	1000	12	6. 5	0. 01	0. 1	FBP
modern hemp (*Cannabis sativa*)	1	1200	12	3. 25	1	6	FBP
modern rapeseed (*Brassica napus*)	1	720	12	3. 25	1	3	FBP
modern adzuki bean (*Vigna angularis*)	1	900	14	6. 5	0. 008	0. 1	FBP
